# The role of angiogenic, anti-angiogenic and vasoactive factors in pre-eclamptic African women: early- versus late-onset pre-eclampsia

**DOI:** 10.5830/CVJA-2012-003

**Published:** 2012-04

**Authors:** Lucinda Govender, Irene Mackraj, Prem Gathiram, Jack Moodley

**Affiliations:** Department of Physiology and Physiological Chemistry, University of KwaZulu-Natal, Durban, South Africa; Department of Physiology and Physiological Chemistry, University of KwaZulu-Natal, Durban, South Africa; Department of Family Medicine, Nelson R Mandela School of Medicine, University of KwaZulu-Natal, Durban, South Africa; Department of Obstetrics and Gynaecology and Women’s Health and HIV Research Group, Nelson R Mandela School of Medicine, University of KwaZulu-Natal, Durban, South Africa

**Keywords:** pre-eclampsia, angiogenic factors, anti-angiogenic factors, sFlt-1, VEGF, PlGF, placental AT1, VEGF, PlGF and sFlt-1 mRNAs

## Abstract

**Abstract:**

The pathogenesis and aetiology of pre-eclampsia (PE) is still unclear. We investigated the role of angiogenic, anti-angiogenic and vasoactive factors in black South African women with early- and late-onset PE. Serum soluble fms-like tyrosine kinase 1 (sFlt-1), soluble vascular endothelial growth factor (VEGF) and placental growth factor (PlGF) levels were determined using the ELISA technique, and placental mRNA expression levels of sFlt-1, VEGF, PlGF and AT1 receptors were determined using real-time PCR.

Serum sFlt-1 levels were significantly elevated and PlGF significantly reduced in early-onset PE compared to the normotensive group. Placental VEGF mRNA expression levels were significantly reduced in the late-onset preeclamptic group compared with the normotensives. The placental mRNA expression of AT1 receptor in the late-onset pre-eclamptic group was relatively raised compared to the normotensives, suggesting hypersensitivity to pressor agents.

We believe that the excess of serum sFlt-1 and reduced VEGF and PlGF levels favour an anti-angiogenic state and endothelial dysfunction leading to PE, and that the aetiology and pathogenesis of early- and late-onset PE differ.

## Abstract

Pre-eclampsia (PE) is a pregnancy-specific syndrome that causes substantial maternal, foetal and neonatal morbidity and mortality worldwide.^1^,^2^ It is characterised by new-onset hypertension and significant proteinuria after 20 weeks of gestation, and the remission of these signs following delivery.

Despite years of research, the exact aetiology of PE remains unknown.^3^ Much is, however, known about its underlying pathophysiology.^4^ It is recognised that PE is a multi-systemic syndrome, affecting several organ systems and is posited to occur in two stages. Stage 1 comprises reduced placental perfusion, which is postulated as the root cause that leads to stage 2; the maternal syndrome.^5^ The cause of the reduced perfusion and the mechanism by which this is translated into the maternal syndrome are still being investigated. The link between stages 1 and 2 may be the key to understanding and eventually treating PE.

It is believed that placental ischaemia during stage 1 may lead to placental production of a soluble factor or factors that cause maternal endothelial dysfunction.^1^,^6^ One such anti-angiogenic factor is soluble fms-like tyrosine kinase 1 (sFlt-1), a soluble vascular endothelial growth factor (VEGF) receptor 1, which has binding sites for soluble VEGF and placental growth factor (PlGF). Therefore, excessive production of sFlt-1 in PE results in a concomitant reduction of free, circulating angiogenic factors, VEGF and PlGF.^7^-^9^

When the maternal endothelium is deprived of these angiogenic factors (VEGF and PlGF) and in the presence of excess anti-angiogenic factors such as sFlt-1, it becomes dysfunctional and leads to the clinical syndrome of hypertension and proteinuria.^1^,^10^ In addition to an imbalance between angiogenic and antiangiogenic factors, the renin–angiotensin system (RAS) has also been implicated in the pathogenesis of PE.^11^-^13^

Most studies on angiogenic/anti-angiogenic factors, however, have been done in high-income countries. Furthermore, there may be racial variations in both the incidence of PE and the clinical features at presentation. For example, the incidence of PE is much higher in South Africa and it has a much more aggressive and rapid clinical course of presentation, leading to significant mortality.^14^ We therefore investigated the role of serum and the placental mRNA expressions of angiogenic and anti-angiogenic factors in African women with early- and late-onset PE in a low- to middle-income setting.

## Methods

Following institutional ethical permission, pregnant women who gave written, informed consent were recruited from the labour ward of a regional hospital in the KwaZulu-Natal province, South Africa. Following enrolment, the participants were grouped as follows: clinically healthy normotensive controls (*n* = 30); chronic hypertensives (*n* = 9) (experimental control); early-onset pre-eclamptics (≤ 27 weeks gestation) (*n* = 10) and late-onset pre-eclamptics (≥ 28 weeks gestation) (*n* = 9).

Definitions: pre-eclampsia was defined as new-onset hypertension (blood pressure 140/90 mmHg) and proteinuria (≥ 2+ on testape) after the 20th week of pregnancy. Chronic hypertension was defined as women with a history of hypertension in the non-pregnant state and who had hypertension at the first antenatal visit prior to 20 weeks’ gestation.

All clinical data were recorded on a structured form and included the highest blood pressure measurement, level of proteinuria, maternal age, parity, gestational age, mode of delivery and neonatal outcomes.

Pre-partum blood samples were obtained from participants who were in early labour. Postpartum central placental samples were obtained soon after delivery of the babies. The blood samples were centrifuged at 4°C at 3 000 rpm for 30 minutes and aliquots of serum samples and placental tissue samples were stored at –70°C until used. All participants with hypertension (chronic hypertensives and pre-eclamptics) were on antihypertensive medication, namely methyldopa.

The quantitative sandwich enzyme immunoassay technique was performed on serum samples to analyse for the levels of sFlt-1, VEGF and PlGF using Quantikine ELISA kits (R & D Systems, Minneapolis, USA). All assays were performed in duplicate according to the manufacturer’s instructions.

RNA was extracted from placental samples using a protocol previously described,^15^,^16^ and synthesis of cDNA was performed using the Bio-Rad iScript cDNA synthesis kit according to the manufacturer’s protocol (Bio-Rad Laboratories (Pty) Ltd). Thereafter, real-time PCR was performed to determine the levels of mRNA expressions of sFlt-1, VEGF, PlGF and AT1 using standard methods.^16^-^18^

## Statistical analysis

All values are expressed as means ± SEM (standard error of mean). Statistical tests were performed using SPSS version 15.0 (SPSS Inc, Chicago, Illinois, USA). A *p*-value of < 0.05 was considered statistically significant. The Kruskal-Wallis test was done to assess for any overall significant differences across the four groups within each variable in clinical data, as well as between the groups for each mRNA gene expression. This was followed by the Mann-Whitney test to determine which groups displayed these differences.

One-way ANOVA was performed to determine if there were significant differences in the serum concentrations of sFlt-1, PlGF and VEGF, and the mRNA expression levels of sFlt-1, VEGF, PlGF and the AT1 receptor among the groups. The Tukey-Kramer multiple comparison test and the Mann-Whitney test compared each group against the other and determined which two groups displayed a significant difference (*p* < 0.05).

## Results

Table 1 shows the demographic and clinical data including neonatal outcomes of all patients. Except for changes in blood pressure and proteinuria, there were no significant differences in any of the parameters among the four groups.

**Table 1:** Demographic Data And Clinical Characteristics Of All Patients

	*Group*				
*Variable*	*Normotensive controls* (n = 29)	*Chronic hypertension* (n = 9)	*Early-onset pre-eclampsia* (n =10)	*Late-onset pre-eclampsia* (n = 9)
Maternal age (years)	28.48 ± 1.19	28.78 ± 1.86	29.88 ± 3.40	28.33 ± 2.05
Parity	2 ± 0.23	2 ± 0.40	2 ± 0.53	1 ± 0.33
Gestational age (weeks)	38.46 ± 0.26 (n = 28)	38.44 ± 0.47	36.38 ± 1.72	37.67 ± 0.73
Blood pressure (mmHg)				
Systolic	118.59 ± 1.79	139.67 ± 3.16	152.75 ± 6.39*	161.78 ± 3.68*^#^
Diastolic	72.24 ± 1.57	91.33 ± 2.74	94.63 ± 5.79*	102.22 ± 3.08* ^#^
HIV status (%)				
Positive	44.83	44.44	25	66.66
Negative	51.72	55.55	62.5	33.33
Unknown	3.45	0	12.5	0
Mass of neonate (kg)	3.24 ± 0.08	3.34 ± 0.17	2.83 ± 0.38 (n = 9)	3.03 ± 0.16

*Significant difference in comparison with normotensive control group (*p* < 0.05). ^#^Significant difference in comparison with chronic hypertensive group (*p* < 0.05).

The body mass of the neonates in the early-onset (2.83 ± 0.38 kg) and late-onset (3.03 ± 0.16 kg) pre-eclamptic groups were lower than those of the normotensive (3.24 ± 0.08 kg) and chronic hypertensive (3.34 ± 0.17 kg) groups. However, these differences were not statistically significant (*p* > 0.05).

**Fig. 1:**
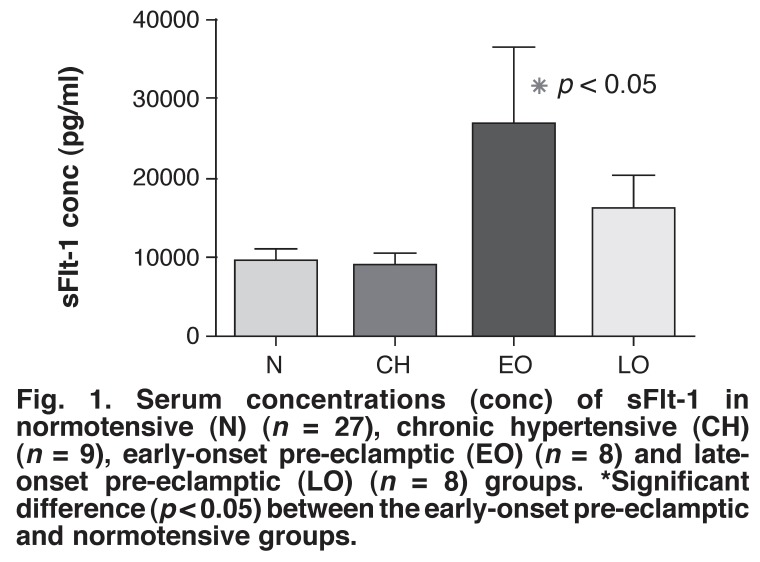
Serum concentrations (conc) of sFlt-1 in normotensive (N) (*n* = 27), chronic hypertensive (CH) (*n* = 9), early-onset pre-eclamptic (EO) (*n* = 8) and lateonset pre-eclamptic (LO) (*n* = 8) groups. *Significant difference (*p* < 0.05) between the early-onset pre-eclamptic and normotensive groups.

The serum sFlt-1 concentrations in the normotensive controls (9 603 ± 1 797 pg/ml) were significantly less than those in the early-onset pre-eclamptic group (26 682 ± 5 482 pg/ml) (*p* < 0.05) (Fig. 1). The levels in the early- and late-onset pre-eclamptic (16 069 ± 4 305 pg/ml) groups were higher than those in the chronic hypertensive group (8 811 ± 2 008 pg/ml) but these changes were not significantly different.

**Fig. 2:**
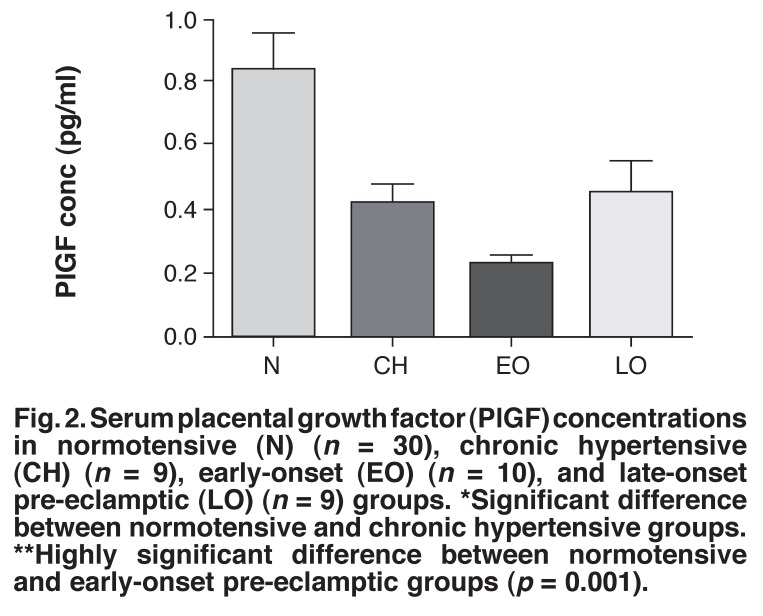
Serum placental growth factor (PlGF) concentrations in normotensive (N) (*n* = 30), chronic hypertensive (CH) (*n* = 9), early-onset (EO) (*n* = 10), and late-onset pre-eclamptic (LO) (*n* = 9) groups. *Significant difference between normotensive and chronic hypertensive groups. **Highly significant difference between normotensive and early-onset pre-eclamptic groups (*p* = 0.001).

The serum concentrations of VEGF in all four groups were below the detectable limit of the assay. Fig. 2 shows that the normotensive group (0.83 ± 0.11 pg/ml) had significantly raised serum PlGF levels compared to the early-onset pre-eclamptic group (0.23 ± 0.031 pg/ml) (*p* = 0.001). Furthermore, there was a significant difference in the levels of PlGF between the normotensive (0.83 ± 0.11 pg/ml) and chronic hypertensive (0.42 ± 0.063 pg/ml) groups (*p* < 0.05) (Fig. 2). In addition, the early-onset pre-eclamptic group had relatively lower serum PlGF levels compared with the late-onset group (0.45 ± 0.103 pg/ml).

The relative placental mRNA expression levels of sFlt-1, VEGF, PlGF and AT1 were compared across the four groups and were normalised to the housekeeping gene, GAPDH. Data are expressed as fold changes.

**Fig. 3:**
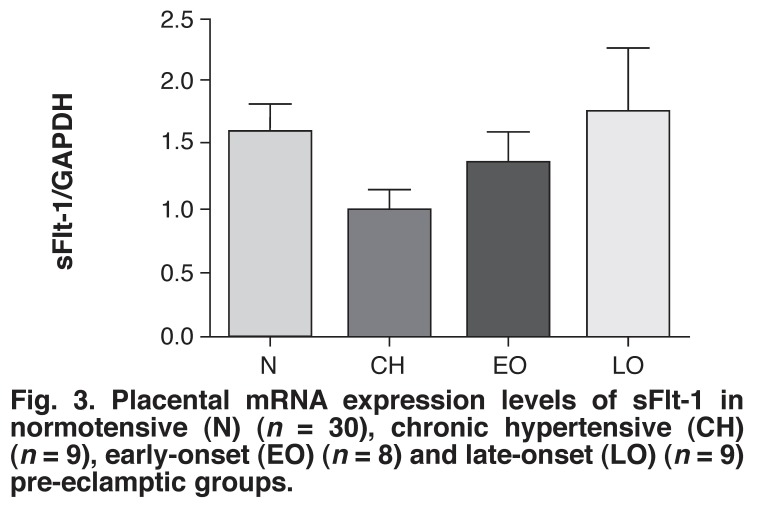
Placental mRNA expression levels of sFlt-1 in normotensive (N) (*n* = 30), chronic hypertensive (CH) (*n* = 9), early-onset (EO) (*n* = 8) and late-onset (LO) (*n* = 9) pre-eclamptic groups.

The highest mRNA expression level of sFlt-1 was found in the late-onset pre-eclamptic group (1.789 ± 0.513 fold) and the lowest level was found in the chronic hypertensive group (1.009 ± 0.162 fold) (Fig. 3). The level of sFlt-1 mRNA expression in the normotensive group (1.62 ± 0.24 fold) was slightly higher than that of the the early-onset pre-eclamptic group (1.401 ± 0.237 fold). There were no significant differences in the sFlt-1 concentrations among the four groups.

**Fig. 4:**
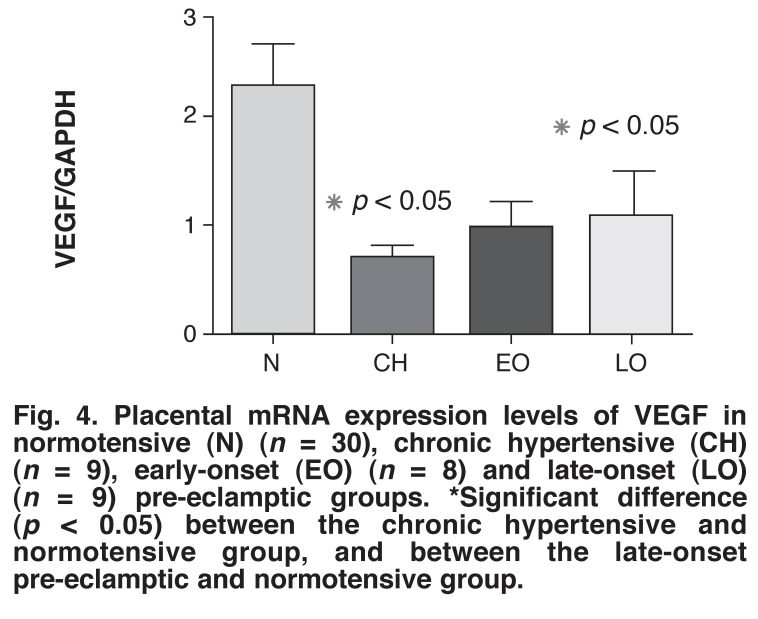
Placental mRNA expression levels of VEGF in normotensive (N) (*n* = 30), chronic hypertensive (CH) (*n* = 9), early-onset (EO) (*n* = 8) and late-onset (LO) (*n* = 9) pre-eclamptic groups. *Significant difference (*p* < 0.05) between the chronic hypertensive and normotensive group, and between the late-onset pre-eclamptic and normotensive group.

The highest level of VEGF mRNA expression was found in the normotensive group (2.261 ± 0.42 fold) (Fig. 4). The VEGF mRNA expression level in the normotensive group was significantly higher than in the late-onset pre-eclamptic (1.059 ± 0.4338 fold) and chronic hypertensive (0.682 ± 0.113 fold) groups (2.14 and 3.24 fold, respectively) (*p* < 0.05). The VEGF mRNA expression level in the normotensive group compared with the early-onset pre-eclamptic group (0.943 ± 0.28) had a *p*-value very close to being significant (*p* = 0.051).

**Fig. 5:**
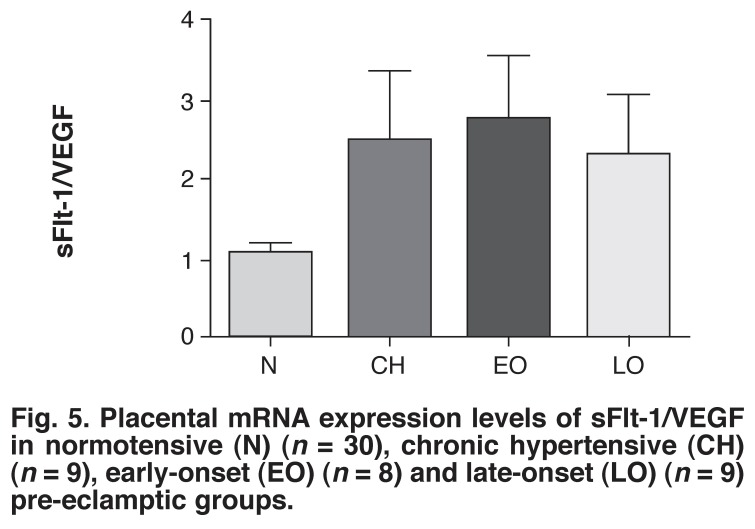
Placental mRNA expression levels of sFlt-1/VEGF in normotensive (N) (*n* = 30), chronic hypertensive (CH) (*n* = 9), early-onset (EO) (*n* = 8) and late-onset (LO) (*n* = 9) pre-eclamptic groups.

As shown in Fig. 5, the ratio of sFlt-1 to VEGF was 2.6-fold higher in the early-onset pre-eclamptic group than in the normotensive group (*p* = 0.055). The ratio in the late-onset pre-eclamptic group and chronic hypertensive group were 2.17- and 2.34-fold greater, respectively than the normotensive group.

**Fig. 6:**
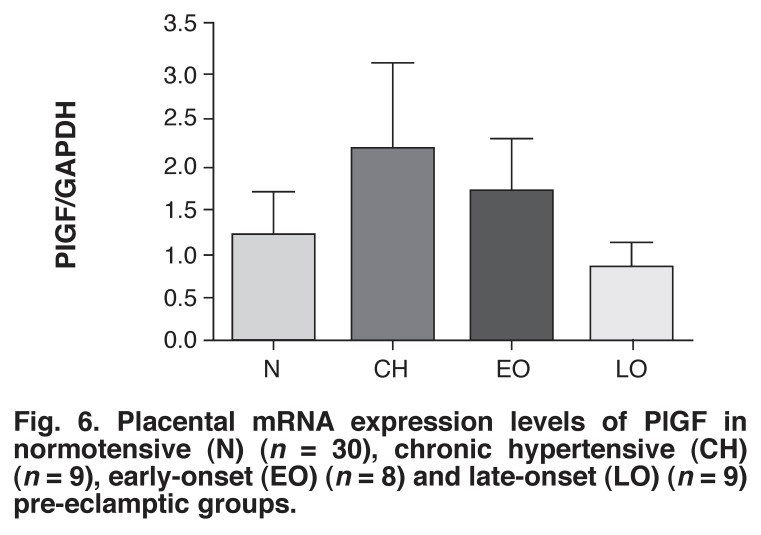
Placental mRNA expression levels of PlGF in normotensive (N) (*n* = 30), chronic hypertensive (CH) (*n* = 9), early-onset (EO) (*n* = 8) and late-onset (LO) (*n* = 9) pre-eclamptic groups.

Although the differences in mRNA expression levels of PlGF among the four groups were not statistically significant, the late-onset pre-eclamptic group had the lowest level (0.81 ± 0.34) while the chronic hypertensive group had the highest level (2.17 ± 0.94) and the latter was 1.80-fold higher than in the normotensive group (1.205 ± 0.4818) (Fig. 6). The early-onset pre-eclamptic group (1.704 ± 0.5854) had a relatively higher level of PlGF expression level than the late-onset pre-eclamptic group but the level was lower than in the chronic hypertensive group.

**Fig. 7:**
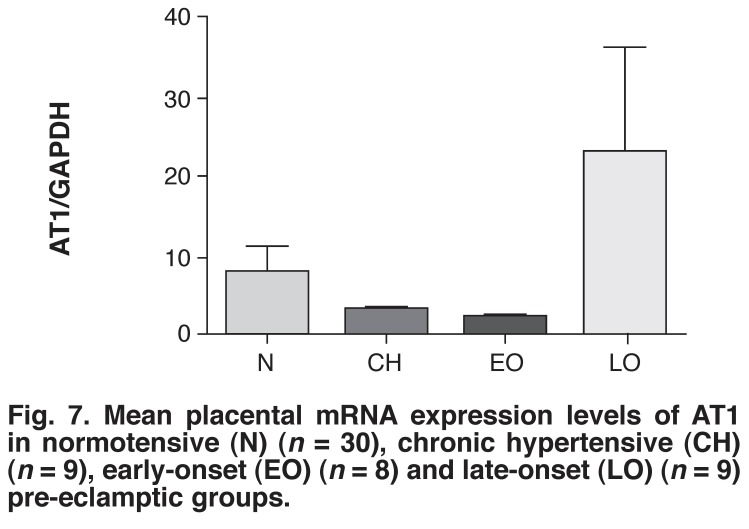
Mean placental mRNA expression levels of AT 1 in normotensive (N) (*n* = 30), chronic hypertensive (CH) (*n* = 9), early-onset (EO) (*n* = 8) and late-onset (LO) (*n* = 9) pre-eclamptic groups.

Among all four groups, the late-onset pre-eclamptic group (23.20 ± 13.36 fold) had the highest level of AT1 mRNA expression (Fig. 7). Furthermore, the normotensive group (7.86 ± 3.34 fold) showed higher AT1 receptor mRNA expression levels than in the early-onset pre-eclamptic (2.13 ± 0.73 fold) and chronic hypertensive (2.63 ± 0.73 fold) groups. Notably, the difference in AT1 receptor mRNA expression level between the early-onset and late-onset pre-eclamptic groups showed a *p*-value very close to significance (*p* = 0.0592 fold). In addition, a positive correlation was found between the AT1 receptor concentration and diastolic blood pressure in the late-onset pre-eclamptic group (*p* < 0.05).

## Discussion

In this study healthy, normotensive, pregnant women served as an appropriate control by representing the physiological changes that occur during uncomplicated pregnancy, which was a point of reference for the pathological conditions of chronic hypertensive pregnancy and PE. Chronic hypertensive pregnancy was a significant reference for the pre-eclamptic groups, indicating that any observations that were common to these groups may be attributed to the high blood pressure that was a common characteristic. Therefore if an observation was made in the pre-eclamptic groups but not the chronic hypertensive group, it could possibly be attributed to the disease pre-eclampsia.

The lower gestational ages in the early- and late-onset pre-eclamptic groups in comparison with the normotensive group may have been as a result of early delivery preventing further complications, as the only known cure for the syndrome is delivery of the baby and placenta.

As expected, the systolic and diastolic blood pressure measurements in the chronic hypertensive and pre-eclamptic groups were significantly higher than in the normotensive controls. Interestingly, the systolic and diastolic blood pressures in the late-onset pre-eclamptic group were significantly higher than in the chronic hypertensive group, indicating that the hypertension in late-onset PE may be more severe than that of chronic hypertension. This may be attributed to the hypertension-inducing effects of sFlt-1, as previously shown in the murine model.^19^

Recent reports suggest that hypertension in response to sFlt-1 may be associated with increased circulating vascular superoxide concentrations and that reactive oxygen species could be involved in mediating the blood pressure response to excessive sFlt-1 during pregnancy. It has been suggested that higher serum sFlt-1 concentrations could play a role in endothelial dysfunction.^10^

The serum VEGF levels in all our groups were below the detectable limit of the assay, which is to be expected in the antenatal period^20^ and has been shown in previous studies.^21^ This could have been due to the mopping-up effect of the excessive levels of sFlt-1 that were observed in this study.

Interestingly, the highest level of VEGF mRNA expression was found in the normotensive group. The level of VEGF mRNA expression was significantly lower in the late-onset pre-eclamptic group, and very close to being significantly lower in the early-onset pre-eclamptic group (*p* = 0.051), compared with the normotensive group. These findings could also have been contributing to the VEGF deficiency in the serum of the pre-eclamptic women. Our study therefore concurs with previous reports showing evidence of reduced VEGF expression in the placentae from pregnancies complicated by PE.^22^-^25^ Such placentae have previously been shown to exhibit morphometric changes such as altered spiral artery remodelling, deficient growth and differentiation of terminal villi, and reduced foetal capillary branching, which could be attributed to reduced VEGF levels.^26^

These studies suggest that VEGF may be implicated in the molecular mechanisms in abnormal placental development and is, therefore an important factor involved in the pathogenesis of PE and its complications. We believe that the relatively high VEGF mRNA expression in the normotensive group compared to the pre-eclamptic groups may indicate that VEGF plays a role in ensuring normal placentation in this group. On the other hand, an immunohistochemical study showed that VEGF levels were significantly higher in placental biopsies of patients with PE than normotensive controls,^27^ while other studies reported no difference in the VEGF mRNA expression levels at term in placentae from pre-eclamptic and normotensive women.^28^,^29^ We postulate that the variations could be due to different techniques used for assessments or to different ethnic populations.

We also observed the circulating level of PlGF to be significantly higher in the normotensive group compared with the pre-eclamptic groups. This finding has also been shown by other workers.^1^,^30^-^32^ It is well documented that during normal pregnancy, the levels of PlGF increase but are dramatically reduced post-partum, since PlGF is produced by syncytiotrophoblast and extravillous cytotrophoblast cells.^33^ However, we did not observe any significant differences in placental tissue mRNA expression levels among the four groups. Further studies may help to shed more light on the control of PIGF levels.

In the present study, the low serum sFlt-1 levels in chronic hypertensive patients corresponded with the low placental sFlt-1 mRNA levels, similar to the normotensive group, whereas in the late-onset pre-eclamptic group, both values were raised. This may indicate that the causes of hypertension in the chronic and late-onset pre-eclamptic groups were not the same and that in the latter group the raised serum sFlt-1 levels could be from other sources.

Furthermore, in the early-onset pre-eclamptic group, the serum sFlt-1 level was significantly greater than the normotensive control group yet no significant differences in the placental mRNA expression levels existed between these groups. This may indicate either that there are other sources of sFlt-1 production in early-onset PE other than the placenta, or that the placental tissue sample may not have been a representative sample of placental villi.

The serum sFlt-1 concentration in the late-onset pre-eclamptic group was relatively less than in the early-onset pre-eclamptic group. Our results support previous studies showing that alterations in the serum sFlt-1 levels are more pronounced in the early-onset pre-eclamptic group than in the late-onset pre-eclamptic group.^34^,^35^ A number of other studies have shown that serum sFlt-1 levels in pre-eclamptic women are increased,^7^,^8^,^36^-^38^ or unchanged,^39^ when compared with normotensive patients. These studies, along with ours, suggest that the placenta is possibly a source of circulating sFlt-1.^38^

In the normotensive group, although the sFlt-1 mRNA expression levels appeared high, the VEGF mRNA expression levels were equally high. Therefore, placental sFlt-1/VEGF mRNA expression ratios were assessed to determine if there was an imbalance between the levels of the angiogenic and anti-angiogenic factors. In the normotensive group this ratio was nearer to 1, indicating that the mRNA expression levels of sFlt-1 and VEGF were similar and therefore balanced.

On the other hand, the sFlt-1/VEGF mRNA expression ratios in early-onset PE were very close to being significantly higher than the normotensive group. The high serum sFlt-1 levels found in the early- and late-onset pre-eclamptic groups corresponded with the high placental sFlt-1/VEGF mRNA expression ratio, suggesting that the imbalance in angiogenic and anti-angiogenic factors begins at the placental level in early- and late-onset PE, promoting an anti-angiogenic state.

Serum sFlt-1 concentrations have been extensively investigated as a key protein that may be involved in the aetiology or as a secondary phenomenon of PE.^40^,^41^ Hypoxia is considered to be the cause of the pathological excessive sFlt-1 production in pre-eclamptic placentae.^1^,^42^

It is believed that this soluble VEGF receptor 1 or sFlt-1 exerts its anti-angiogenic functions by binding to and inactivating VEGF and PlGF. This is the basis of the antagonistic relationship between sFlt-1 and VEGF. The authority of sFlt-1 lies in the fact that it is capable of upsetting the delicate equilibrium, and tipping the balance towards the anti-angiogenic state in PE. This would, in turn, have a negative effect on the vasculature since VEGF plays crucial roles in important processes such as the control of angiogenesis. Notably, autocrine VEGF has a trophic effect on the endothelium, distinct from its control of angiogenesis. By inhibiting this effect, elevated sFlt-1 levels may lead to systemic endothelial cell dysfunction, which is the hallmark of the maternal stage of PE.^43^,^44^

Another meaningful relationship has been demonstrated between sFlt-1 and the RAS.^45^ In addition to hypoxia causing increased sFlt-1 production, recent studies have revealed that AT1 autoantibody induces excess sFlt-1 production, secretion and impaired angiogenesis in PE through undefined mechanisms.^11^,^46^ The AT1 autoantibody and hypoxia-induced overproduction of sFlt-1 could activate a dangerous positive feed-forward cycle wherein high sFlt-1 levels severely inhibit angiogenesis and exacerbate the placental hypoxia observed in PE, which subsequently causes an increase in placental sFlt-1 production.^40^,^47^ Furthermore, recent studies have shown that AT1 receptor signalling regulates the genes encoding for proteins associated with angiogenesis, such as sFlt-1.^47^

In this study the AT1 receptor expression levels were relatively lower in the normotensive, chronic hypertensive and early-onset pre-eclamptic groups than in the late-onset pre-eclamptic group. This can be expected in the normotensive group since in normal pregnancies there is a reduced response to vasopressors.^48^ However, AT1 receptor mRNA expression level in the late-onset pre-eclamptic group was very close to being significantly higher than in the early-onset pre-eclamptic group (*p* = 0.059), suggesting that the RAS may be implicated in the pathogenesis of PE in this group. This may also indicate that late-onset PE is a different disease from early-onset PE and, therefore may actually be a disease that has a closer association with the RAS than the angiogenic/anti-angiogenic theory, as is the case in early-onset PE.

The raised AT1 receptor mRNA expression level in late-onset PE may be due to raised AT1 autoantibodies in this group.^40^,^49^ Furthermore, we also found a significant correlation between AT1 receptor mRNA levels and diastolic blood pressure in the late-onset pre-eclampsia group (*p* < 0.05). Unfortunately, the factors that activate the AT1 receptors, including angiotensin II and the AT1 auto-antibodies were not quantified in this study.

Further studies using a larger sample size and measuring AT1 receptor mRNA expressions, AT1 autoantibodies and blood pressure in these two groups are necessary to prove this hypothesis. Interestingly, a recent study showed that the increase in AT1 autoantibodies might represent a better marker for late-onset PE, whereas sFlt-1 is a better marker for early-onset PE.^50^

To the best of our knowledge, this is the first time it has been shown in black South African women that placental AT1 gene expression levels were relatively elevated in late-onset PE compared with the early-onset pre-eclamptic, normotensive and chronic hypertensive groups. In another study conducted on black South African women, it was shown that the common RAS polymorphisms could not be used to predict PE.^51^

It has previously been shown that the blunted response to angiotensin II during normal pregnancy is lost in pre-eclamptic patients, who show a hypersensitivity to vasopressors.^48^ This may imply that there is no quantitative increase in vasopressors, but rather an increase in sensitivity to the existing quantity of vasoactive factors present in pre-eclamptic women. Our study provides proof of this by showing elevated expression of the AT1 receptors in late-onset pre-eclamptic patients compared to the normotensive controls, chronic hypertensive and early-onset pre-eclamptic groups, thereby accounting for the heightened sensitivity to the powerful vasoconstrictor angiotensin II, which is in accordance with previous studies.^52^,^53^

Most studies on PE have been conducted on Caucasian participants, and there is a paucity of information on other ethnic groups. It is well documented that socio-demographic factors such as race have an influence on the incidence of PE in a population.^54^ The high incidence of PE in South Africa as well as the increased risk of mortality associated with PE in black women stress the need for research on homogenous population groups.

We recommend that future research should also incorporate the investigation of the heterodimerisation of the AT1 and bradykinin B2 receptors in black South African women. Furthermore, the interplay between the angiogenic/anti-angiogenic pathway, RAS and other theories, such as that of oxidative stress and the role of the inflammatory system in PE, should be explored.

Two of the limitations of this study were the small sample size that affected the statistical analysis, and the fact we did not measure serum levels of angiotensin II and AT1 autoantibodies.

## Conclusion

The findings of this study lead us to conclude that the aetiology and pathogenesis of early- and late-onset PE are, to some extent different, especially in our population sample. Our data demonstrate that while a predominance of an anti-angiogenic state may be an important role player in both early- and lateonset PE, in the latter, AT1 receptor up-regulation and the RAS could also play a role.
